# Three Dimensional Fluorescence Imaging Using Multiple Light-Sheet Microscopy

**DOI:** 10.1371/journal.pone.0096551

**Published:** 2014-06-09

**Authors:** Kavya Mohan, Subhajit B. Purnapatra, Partha Pratim Mondal

**Affiliations:** 1 Department of Instrumentation and Applied Physics, Indian Institute of Science, Bangalore, India; 2 Applied Photonics Initiative, Indian Institute of Science, Bangalore, India; Tufts University, United States of America

## Abstract

We developed a multiple light-sheet microscopy (MLSM) system capable of 3D fluorescence imaging. Employing spatial filter in the excitation arm of a SPIM system, we successfully generated multiple light-sheets. This improves upon the existing SPIM system and is capable of 3D volume imaging by simultaneously illuminating multiple planes in the sample. Theta detection geometry is employed for data acquisition from multiple specimen layers. This detection scheme inherits many advantages including, background reduction, cross-talk free fluorescence detection and high-resolution at long working distance. Using this technique, we generated 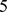 equi-intense light-sheets of thickness approximately 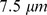 with an inter-sheet separation of 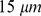. Moreover, the light-sheets generated by MLSM is found to be 2 times thinner than the state-of-art SPIM system. Imaging of fluorescently coated yeast cells of size 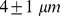 (encaged in Agarose gel-matrix) is achieved. Proposed imaging technique may accelerate the field of fluorescence microscopy, cell biology and biophotonics.

## Introduction

3D fluorescence imaging is fast becoming important for accessing disease progression in clinical trials. Often 3D volume block is constructed from point-by-point or at best plane-by-plane scanning of the specimen. These techniques suffer from three main limitations: (1) Prolonged scanning process that consumes most of the precious imaging time, (2) Suffer from photobleaching over long scans thereby reduces signal-to-noise ratio (SNR) and, (3) It lacks the ability to simultaneously visualize multiple specimen layers. 3D imaging has the advantage that it can perform in-vivo monitoring of dynamical events in cellular compartments for prolonged time especially when they are carrying out key biological processes with the best available spatio-temporal resolution. The other limitation of the existing imaging system is its inability to follow rapidly occurring biological events (timescale: few 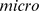-to-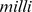 seconds) such as, the release of 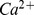 ions during muscle contraction (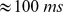) [1] [2], protein folding kinetics [3] [4] [5], and dynamical behavior of Golgi units in living plant cells [6]. Super-resolution techniques have often been used to visualize these nanoscale dynamics. Recently, Liu et al., used a two-color stimulated emission depletion (STED) microscope to show that, a Rab3-interacting molecule (RIM) binding protein is essential for rapid release of neurotransmitter with a resolution of 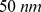 [7]. Kner et al., has shown high-speed structured-illumination microscope for imaging tubulin and kinesin dynamics in living Drosophila melanogaster S2 cells [8]. Many variants of super-resolution imaging techniques have shown promising development in the last decade [9] [10] [11] [12] [13] [14]. Most of these techniques employ point-by-point illumination and detection. A relatively recent development in fluorescence microscopy is light-sheet based planar illumination technique popularly known as single plane illumination microscopy (SPIM) [15] [16]. This technique employs a sheet-of-light for illumination and an entire plane can be imaged. This technique was initially introduced by Voie et al. [17] and vastly improved by Stelzer’s group [18] [19] [16]. Over the last few years, there have been many variants such as, ultramicroscopy [20], objective coupled planar illumination microscopy (OCPI) [21], extended light-sheet microscopy [30] and individual molecule localization SPIM (IML-SPIM) [31]. Of-late confocal detection techniques were employed for enhancing contrast and improving the signal-to-noise ratio in light sheet microscopy [32] [33]. Moreover, Bessel beam has been successfully used to generate thinner light-sheets [34] and to improve the penetration depth in large scattering media [35] [36].

Efforts have been made to reduce the scanning time using optical techniques. One such technique involves the splitting of fluorescence light into multiple parts after being collected by the objective lens and fed to multiple detectors. They have reported imaging upto a maximum of four planes [22]. A similar technique based on splitting the output fluorescence by beam-splitters was used to quantize the temporal resolution of 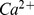 signals [23]. A relatively faster technique was proposed by Abrahamsson et al. [24], where, multifocus grating is used in conjunction with chromatic corrected grating and prism. They have reported simultaneous excitation of nine focal planes. In a similar study, Dalgarno has achieved multi-plane imaging and 3D particle tracking by using optical elements, such as, a diffraction grating in the detection path of the imaging system [25]. Recently, we have proposed a new microscopy technique based on the generation of multiple excitation-spots (MESO microscopy) that has the ability to excite multiple specimen layers [26] [27]. These techniques have two primary limitations: they often carry optical abberations (spherical abberations, that occurs primarily due to refocusing and chromatic abberation due to multi-color imaging), and they are point-by-point based scanning techniques. Moreover, refocusing as required by these techniques has its own limitations.

In this article, we take a step further to aid 3D volume imaging using multiple light-sheets without employing point-by-point (CLSM, STED and TPE) or slice-by-slice scanning (SPIM, IML-SPIM) [28], [29] [31]. This microscopy technique is termed as multiple light-sheet microscopy (MLSM). This is made possible by spatial filtering technique. An appropriate spatial filter is placed before the cylindrical lens that results in interference at and near the focal plane, thereby generating multiple light-sheets. These light-sheets were successfully used to image multiple planes of a tissue-like gel-matrix containing fluorescently-coated yeast cells.

## Results

We demonstrate the generation of multiple light-sheets for simultaneous visualization of multiple specimen layers. The whole imaging system is computationally simulated, experimentally demonstrated and the results are discussed. Utilizing the fact that, fluorescence emission is isotropic, theta detection is employed to cut-off the illumination light [37] [38]. In general, the axial resolution of conventional microscopes is inferior to the transverse resolution and this fact adversely affects their ability to obtain high-resolution 3D images. In an orthogonal detection geometry, the axial resolution of the system is essentially determined by the lateral resolution of the detection sub-system. This improves the axial resolution of the overall imaging system. Finally, the results of the proposed MLSM technique is compared with the state-of-art SPIM system.

**Figure 1:**
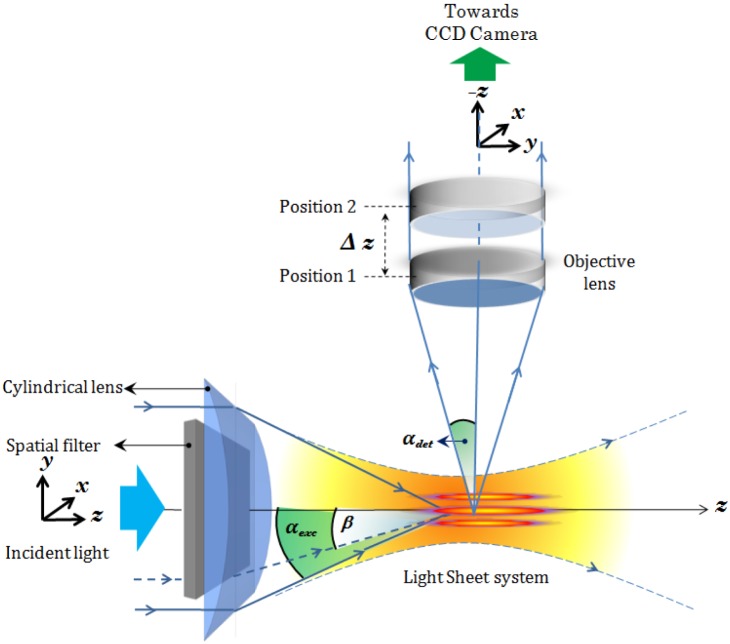
Schematic diagram of the complete imaging system. The illumination sub-system employs spatial filter at the back-focal plane of the cylindrical lens for generating multiple light-sheet pattern. The detection sub-system is essentially a theta-detection system (orthogonal detection) with a fine scanning ability along 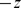 axis. Position 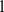 and 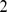 indicate two scan positions of the detector sub-system.

**Figure 2:**
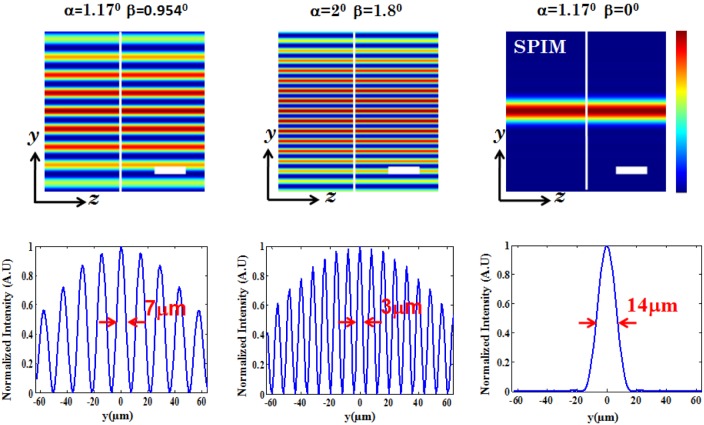
Illumination PSFs obtained from computational simulation. The study was carried out for two configuration, small numerical aperture (aperture angle, 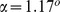, stop angle, 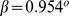), and large numerical aperture (aperture angle, 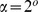, stop angle, 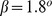). For comparison, the illumination PSF of the state-of-art SPIM system (aperture angle, 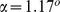, stop angle, 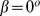) is also shown. Scale bar is 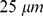.

**Figure 3:**
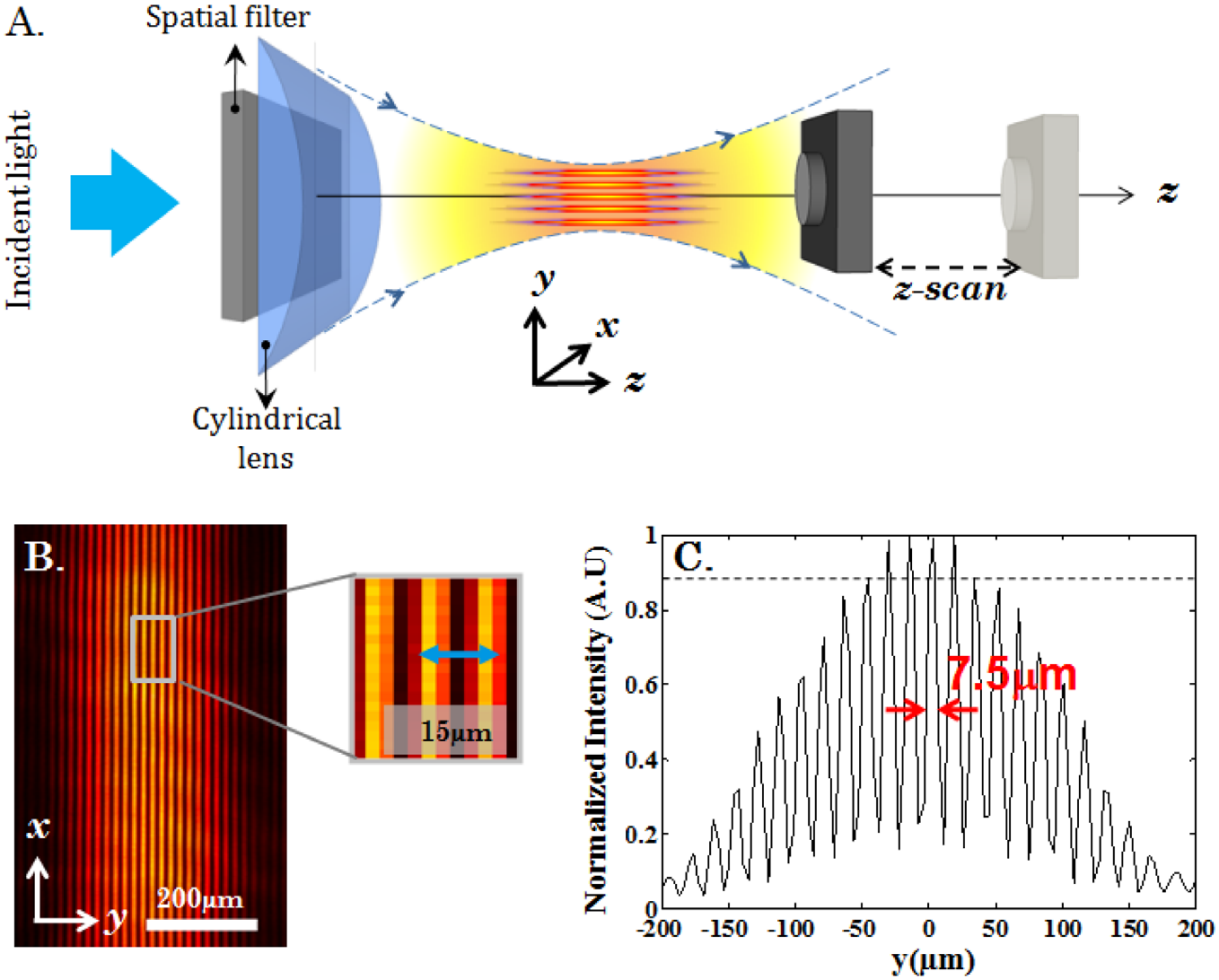
Experimental generation of multiple light-sheets and characterization. (A) Schematic diagram of the experimental setup used for characterizing the illumination PSF. (B) Experimentally obtained transverse profile of the multiple light-sheet system. (C) Intensity plot of the experimentally obtained illumination PSF along y−axis. The inter sheet separation is found to be 15 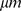 whereas the FHWM of each sheet is 7.5 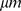. The dotted line indicate 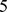 prominent light-sheets with relative intensity 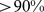.

The schematic diagram of the proposed multiple light-sheet microscopy (MLSM) system is shown in Fig. 1. In general, the system can be broadly splitted into two independent optical configurations: Excitation sub-system and Detection sub-system. Our primary goal is the generation of multiple light-sheet. This will enable simultaneous monitoring of the specimen with reduced photobleaching and may expedite volume imaging. We employ theta detection system which embodies many advantage over the existing detection techniques. Light of wavelength 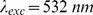 is allowed to pass through the spatial filter, thereby resulting in a structured wavefront. Fig. 1 shows the excitation and detection sub-system along with the imaging parameters. The spatial filter in the illumination sub-system is described in terms of stop angle 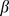. The structured light is then focused by the cylindrical lens which performs 1D Fourier transform, thereby resulting in distinct field distribution at and near the focal plane. It may be noted that, it is the 1D focusing property of cylindrical lens that results in the formation of light-sheets transverse to the optical z−axis. The illumination PSFs of the proposed imaging modality is obtained using eqns. (2) and (4) for small (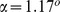) and large (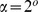) aperture angles respectively. Computational simulation reveal the generation of multiple light-sheets for varying aperture angle. The stop angle of the spatial filter is purposefully chosen to fill 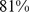 of the aperture angle of the cylindrical lens. Multiple light-sheets along the detection axis (i.e, 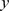
−axis) is evident in Fig. 2. The intensity of the side-lobes are purely determined by the stop angle (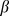-parameters) of the spatial filter. This facilitates simultaneous excitation of multiple specimen layers. From the intensity plots (Fig. 2), it is evident that the central lobe and the first few side lobes on either sides are equally intense. Thereafter, the intensity falls off gradually on both the sides. This is due to the fact that, the 1D Fourier transform (performed by the cylindrical lens) of a rectangular window function (spatial filter) is a 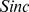 function (intensity distribution at focus). The multi-sheet pattern also suggests a substantial reduction in light-sheet thickness. At low NA (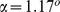), computational study show the sheet thickness of about 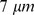 (see, Fig. 2, first column), whereas, the sheet thickness is found to be 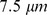 experimentally (see, Fig. 3 (C)). Comparatively MLSM light-sheets are almost 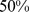 thinner than that of light-sheets obtained using state-of-art SPIM system (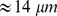). However in a real thick sample, the out of focus contribution may be much higher than the in-focus signal due to non-uniform illumination. So, the contrast of the proposed technique may not be much higher than that of a conventional widefield microscope. It is further observed that, the dimension of light-sheet decreases substantially with an increase in the semi-aperture angle (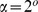) as seen in Fig. 2. Overall, this suggests that, one can reliably scan 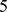 layers of the specimen in a single shot with thinner light-sheets. A comparison with state-of-art SPIM system (corresponding to 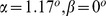 in Fig. 2) is also shown. The ability to control the number of light-sheets and its thickness adds another dimension to 3D fluorescence imaging and is a step closer to realize volume imaging.

The schematic diagram of the optical setup for the experimental determination of the field distribution is as shown in Fig. 3A. A camera was placed directly near the focus of the cylindrical lens and scanned across the focal plane. The light-sheets were characterised over a range of 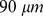 from 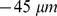 to 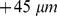 along the 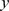
−axis. This spatial region embodies 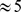 prominent light-sheets (with intensity 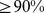 of the peak) as evidenced by the intensity plots shown in Fig. 3C. that extends over a total distance of 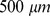 (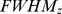). Individual light-sheets have a thickness of 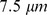 (FWHM) and the inter-sheet separation is 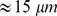. The experimental data suggests that in-principle 3D imaging can be performed at-best by 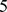 prominent light-sheets.

**Figure 4:**
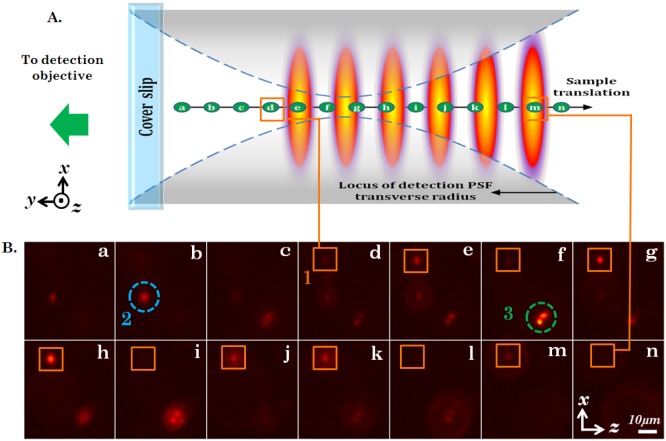
Fluorescently coated yeast cells, encaged in gel matrix, imaged by multi-sheet excitation and orthogonal detection. (A) Cartoon depicting various positions (a–n) of a particular yeast cell as the sample is translated with respect to the detection focal region and the multi-sheet system. (B) Image sequence captured from the orthogonal detection arm. Each frame is spatially separated by 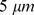 along the y−axis. The primary region of interest is marked by the number ‘1′ and enclosed by an orange square in frames (d)–(n) and the cell within it exhibits variation in overall intensity, indicating the presence of multiple light-sheets. This can be understood clearly when compared with the cartoon in B. Additionally, the images of the cell defocus far from the detection focus. The cells ‘2′ and ‘3′ encircled in blue and green respectively, also exhibit similar intensity variations and defocusing but are situated at different 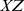-planes.

We performed imaging of fluorescently-coated yeast cells using the proposed imaging system. The 3D specimen was essentially fluorescently-coated yeast cells encaged in a Agarose gel-matrix. We employed an theta detection system (orthogonal detection to the illumination sub-system). The detection arm consists of a 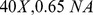 objective lens that collects fluorescence light from the specimen. Subsequently, the light was filtered by a long-pass filter (Thorlabs FEL550, 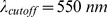) to remove scattered incident light and focused to the CCD camera (Jenoptik, MFCool) by a tube lens (focal length 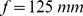). The focus of the detection objective was aligned to coincide with the multisheet pattern. We performed imaging by both translating the specimen as well as translating the detection sub-system. In the first case, the sample was translated along the axis of the detection arm (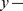
axis) in steps of 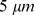 using a high precision micrometer translator, and the data from individual light-sheet was recorded. Fig. 4A shows the cartoon demonstrating the experimental details and the data acquisition procedure. The sample (gel-matrix) is mounted on the coverslip and exposed to the multi-sheet illumination pattern. The alphabets 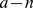 indicates the position of a particular specimen layer. Fig. 4B shows the images obtained by translating the specimen. One can clearly see several single yeast cells throughout the excited specimen layers. We have marked three yeast cells and numbered them as 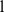, 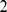 and 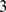 respectively. Specifically, we observe two effects: (1) Due to the alternate dark and bright regions, the intensity modulation occurs as the sample is translated, and (2) a defocusing effect of high NA detection objective as the specimen layer moves away from the common focus (see, detection PSF in Fig. 4A). These two effects are evident from the frames (a–n). Particularly, note the cell marked as 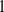 (see, orange box), produces strong intensity modulation as it pass through bright (high intensity) regions of the excitation pattern. Frames, 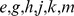 have high intensity as compared to frames 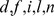 for cell marked as 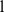. The other cells marked 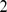 and 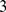 also exhibit similar effect particularly 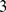 indicating yeast-budding. This demonstrates simultaneous 3D multi-layer illumination capability of the specimen planes employing multiple light-sheet pattern with a reliable theta-detection system. Additional optics and control can be brought-in to automatize the imaging technique.

**Figure 5:**
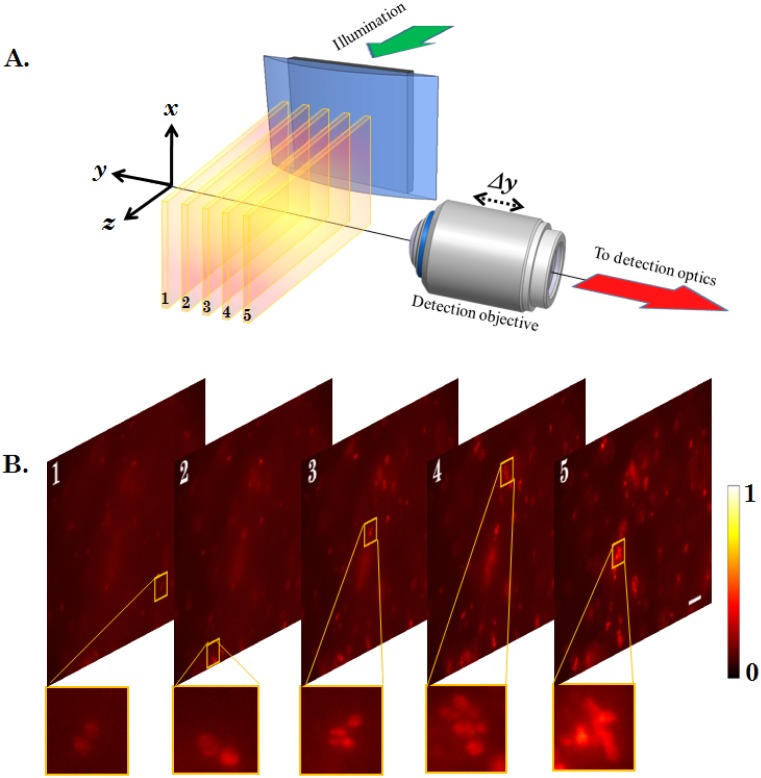
Demonstration of multi-plane imaging capability. (A) Schematic depiction of the experimental setup (not to scale). The near focal region of the cylindrical lens is magnified to show the multiple light-sheets. The orthogonal detection arm is traversed along the 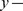
axis to focus on the different light-sheets. The numbers 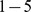 figuratively depicts five light-sheets from which experimental images were obtained. (B) Experimental images corresponding to different planes illuminated by the light-sheets. The sample used was fluorescently coated yeast cells encaged in agar gel matrix. Each image is parallel to the 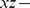 plane and contains images of yeast cells in focus and defocussed images of cells illuminated by other light-sheets. Some of the in-focus cells are marked by the orange boxes and those regions are zoomed and presented below each such image. The distance between each image (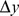) is approximately 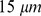 and the scalebar represents 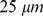.

Next, we obtain sectional images of specimen layers using the proposed MLSM system by translating the detector sub-system instead of sample translation. The schematic diagram illustrating the illumination and detection schemes is shown in Fig. 5A. Fine translation of detection was achieved by a high-precision z-translator. The corresponding sectional images are shown in Fig. 5B. As we translate the detection arm along 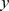
−axis, we observe a series of in-focus and out-of-focus image planes. In-focus planes are observed as a result of intersection of a particular light-sheet and detection PSF. Since light-sheets are separated by approximately 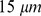, the background is substantially reduced. This shows that 3D imaging can be performed using the proposed multiple light-sheet illumination technique.

## Discussions

SPIM has created a lot of interest in the field of fluorescence microscopy and imaging. This technique overcomes some of the key limitations over the existing imaging systems (widefield, confocal, two-photon and super-resolution microscopy). Some of the advantages of light-sheet based techniques are: low photon flux for prolonged imaging with minimal photobleaching effects, single-shot technique to obtain optically sectioned images and better temporal resolution. This technique continues to expand with the incorporation of super-resolution in SPIM [31]. In this article, we further develop this technique to add the third dimension by employing spatial-filtering technique in a light-sheet based fluorescence microscopy.

The ability to visualize multiple specimen layers add another dimension to the light-sheet based imaging system. The existing point-by-point and plane-by-plane based scanning techniques are limited by scanning time and requires complex arrangement of optical components. This brings in a lot of error due to mechanical parts (galvanometric scanning mirror) apart from the high cost. This further requires a lot of expertise and frequent alignment for accurate functioning. Although CLSM, STED and SPIM have progressed rapidly, but the need of single-shot multi-layer visualization in fluorescence microscopy is essential for further development. By employing spatial filter at the back-aperture of the cylindrical lens, an array of light-sheets can be generated that can illuminate the entire volume in a single-step. The pros of proposed MLSM technique are: (1) The ability to monitor and visualize specimen for long time, (2) reduced photobleaching due to multiple light-sheet (low-photon flux) illumination and, (3) total cut-off of scattered light (reduces background). The disadvantage of the proposed technique is that the spatial filtering blocks a large portion (almost 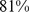) of the incident light. This prohibits judicious utilization of light. This can be improved upon by using donut shapped beam that has its maximum energy at the pheriphery rather than at the center, as is the case for Gaussian beam. To ensure sufficient intensity for imaging fluorescently-coated yeast cells, we have jacked-up the laser intensity. In future, we plan to modify the technique to minimize the photon budget.

The experimental realization shows the generation of multiple light-sheets. Specifically, a total of 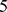 prominent light-sheets has been observed along with auxiliary light-sheets. We could use only 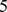 light-sheets for imaging encaged yeast cells. The thickness of individual light-sheet is about 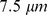 which closely match the theoretically predicted values. The spacing of light-sheets is about 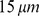 that is beneficial for imaging large specimens. The MLSM light-sheet is found to be 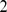 times thinner than that of SPIM system. This reduce cross-talks from the nearby illuminated planes. We could scan an area of 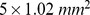 (for 5 layers) using the proposed multisheet imaging technique. In future, we plan to increase the number of light-sheets and expand individual light-sheets for large field-of-view. For simultaneous multi-layer detection, one can employ a detection system that can split the fluorescence coming from different planes and diverts it to multiple detectors [25]. Moreover, it may be noted that every part of the specimen lying inside one of the light sheets will emit fluorescence when the real sample is illuminated using the multiple light sheets. However, only one light sheet can be in the depth of focus of detection optics. The fluorescence from all other planes will only contribute as out-of-focus background, thereby reducing image contrast to the level of a standard wide-field microscope. This effect can be seen in Fig. 4 and Fig. 5. For better selectivity, a confocal detection can be employed to reduce out-of-focus background. Proposed MLSM imaging technique may facilitate long-time monitoring of 3D specimen. Other exciting development is to integrate Bessel beam for depth imaging using spatial filtering [39]. This may further trigger new application in fields as diverse as volume imaging, biophotonics and cell biology.

## Methods

### Theory behind the Multiple Light-sheet Generation

Consider a cylindrical lens with its primary axis along 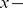 direction. In cylindrical coordinates (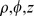), the electric field components at the focus of a linearly polarized light illumination (polarization angle 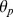 with 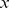
−axis) with profile 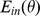 is given by [44],
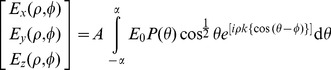
(1)where, 
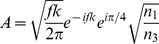
, and 
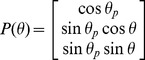
. Here 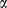 is the semi-aperture angle of the lens defined by it’s numerical aperture and 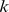 is the wavenumber in the image space. The terms 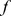, 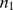 and 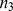 denote the focal length, refractive index of object and image space respectively while the radial distance from the 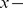
axis and the polar inclination are denoted by, 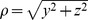 and 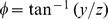 respectively. By introducing an amplitude transmission function 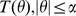 (that of spatial filter), Eq.(1) modifies to,



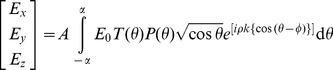
(2)We employ a binary spatial filter for which the transmission function 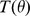 is given by,
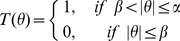
(3)where, 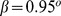 and 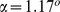.

In terms of actual distance along 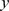
−axis, the spatial filter can be expressed as,
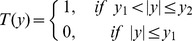
(4)where, 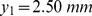 (half-width of spatial filter) and 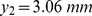 (full beam-width).

### Experimental Generation and Characterization of Multiple Light-sheets

For this study, we assumed plane-wave illumination and compute the field distribution (at and near the focus) of the MLSM system. The computational study was carried out on a spatial dimension of 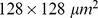 along 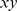-plane and 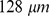 along z−axis. A monochromatic excitation source of wavelength, 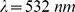 and objective lens (air) of semi-aperture angles ranging from 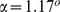 to 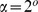 was used. To evaluate the system PSF, the parameters were chosen that are suitable for observing Alexa Fluor 532 dye fluorophores (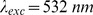, and 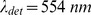) as a probe [40] [41].

To generate multiple light-sheets, we used spatial filtering technique. Due to the geometry of the cylindrical lens that diffracts light along y−axis only, the optical mask was designed to create a spatial filter along that dimension. During experimentation, we have incorporated the fact that, the beam is Gaussian. It may be reminded that, cylindrical lens produces a line focus rather than point focus. The back aperture of the plano-convex cylindrical lens (Thorlabs, 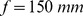) was subjected to a specially fabricated optical mask of width 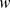 that corresponds to the desired stop angle 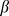 as determined by the computational study. They are related by the simple relation, 
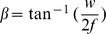
 (see, Fig. 1). The mask was aligned parallel to the cylindrical axis (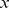
−axis in Fig. 1). Care must be taken to align the mask as small mis-match may result in asymmetric light-sheets. The optical mask was illuminated by an expanded Gaussian beam of diameter 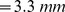 ((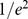) value) (Excel laser, 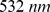, vertical polarization). A CMOS camera (PointGrey-Chameleon) was employed to directly measure the field distribution. The camera was mounted on a linear micrometer translator (Holmarc, TS-65-Mu10). The translator was moved along the optical 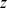
−axis to take multiple snap-shots of the field distribution with a step-size of 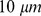. Multiple ND filters were used to protect the sensor from high power.

### Spatial-filter Fabrication and Characterization

At the heart of the system is the the specially designed spatial filter. The filter used in this experiment was fabricated based on the stop angle. Computational studies were performed to determine the parameters of spatial filter. Specifically, varying width (w)/stop angle (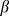) were used before fixing, 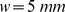 (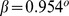) that gives the desired results. In this experiment, we have used 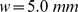 that correspond to a stop angle of 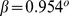. The parameters were chosen based on rigorous computational study. The transmission characteristics of the spatial filter is as shown in the supplementary 2, Fig. 2. This spatial filter gives the best inter light-sheet spacing and its thickness. For the chosen filter, the sheet spacing is 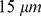 with a thickness of 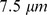.

### Sample Preparation

#### Materials

Dimethyl sulfoxide (DMSO) were obtained from Sigma-Aldrich. The fluorescent dye NHS-Rhodamine (MW: 528, Ex/Em wavelength: 552/575) and fluorescent dye removal columns were purchased from Thermo Scientific. Agar powder was acquired from Sd Fine Chemicals. An optical filter with a cutoff at 550 nm was purchased from Thorlabs for blocking the incident light. Poly(styrenesulfonate sodium salt) (PSS; MW = 70,000), poly(allylamine hydrochloride) (PAH; MW = 15,000), Ammonium bicarbonate (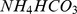), Calcium Nitrate(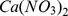) and Sodium chloride (NaCl, 0.5 M) were purchased from Sigma-Aldrich.

#### Preparation of fluorescent conjugate polymer

To prepare the conjugate polymer, we have followed the procedure reported in Ref.[42] and Ref.[43]. First step consists of mixing a PAH solution (1 mg/ml) prepared in double-distilled water, with the dye dissolved in DMSO (10 mM), in a 1:15 (w:w) ratio. The mixture was then placed in the incubator for some time and the darkness was maintained overnight with continuous gentle stirring to avoiding photobleaching. Subsequently, the resulting PAH-NHS Rhodamine was purified using fluorescent dye removal columns.

#### Encaged fluorescently-coated yeast cells

For the preparation of Agarose gel-matrix, 200 mg of Agar powder was dissolved in 20 ml distilled water and heated at 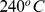 for 10 minutes. The melted agar gel was then cooled to 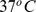. 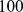 ml of melted agar gel was added to 50 ml of fluorescently coated yeast cells with constant stirring. After solidification of the gel, it was cut and shaped as a sheet of thickness 3 mm. The gel sample was then carefully sandwiched between two coverslips and a holder is fabricated to hold it. Special care was taken to obtain a smooth surface at both the surface facing excitation light-sheet and the surface facing the detector.
